# A step change model analysis of the establishment of pill testing in one Australian jurisdiction

**DOI:** 10.1186/s12954-023-00907-6

**Published:** 2023-11-30

**Authors:** David Caldicott, Toni Makkai, Malcolm McLeod, Stephanie Tzanetis, Gino Vumbaca

**Affiliations:** 1 Medical School, Australian National University, Florey Building 54 Mills Road, Canberra, ACT 2601 Australia; 2 Centre for Social Research and Methods, Australian National University, RSSS Building, Canberra, ACT 2601 Australia; 3 Research School of Chemistry, Australian National University, Canberra, ACT 2601 Australia; 4 Harm Reduction Australia, 17 Glenugie St, Maroubra, NSW 2035 Australia

**Keywords:** Harm reduction, Pill testing/drug checking, Service delivery, Policy change

## Abstract

This paper applies the theory of change model (Kotter in Harv Bus Rev 2:59–67, 1995; Moore et al. in Viet Nam J Public Health 1(1):66–75, 2013) to describe the pathway that lead to Australia’s first pill testing/drug checking services in Canberra, in the Australian Capital Territory. The paper takes each step of the model and illustrates the key activities that largely occurred over an approximately 24 month period resulting in the service being operational on 29 April 2018. The paper demonstrates that leadership, advocacy and activism are key components, alongside evidence, to bringing about public policy change. It provides a unique insight to the extensive efforts undertaken to achieving the first legally sanctioned pill testing at festivals in Australia and provides a positive case study for those seeking to introduce contested harm reduction services in the drug and alcohol field.

*“Never doubt that a small group of thoughtful, committed citizens can change the world. Indeed, it is the only thing that ever has…”*

*Margaret Mead*

By tradition, medicine is a conservative profession, and often slow to change in policy and practice, even in the face of evidence. Due to the potential risks to patients, new applications and services are often subject to extensive clinical trials and government regulation, resulting in long lead times before evidence is put into day-to-day practice. Yet many of the great medical challenges—obesity, diabetes, vaccine and hygiene adherence, mental health and drug use—are inherently social and values-based, requiring leadership, advocacy and activism to achieve full ‘knowledge translation’. These aspects of medical care are relative *arrivistes* in many health and medical curricula in Australia.

This paper describes the pathway that lead to Australia’s first pill testing/drug checking services in Canberra, in the Australian Capital Territory (ACT), to illustrate how change in a socially and politically contested space requires leadership, advocacy and activism, alongside evidence. Advocates for pill testing in Australia have been vocal about the benefits [14], based on evidence emerging out of Europe since the early 2000s [23], against fierce opposition at times, but it was not until 2018 that the first government sanctioned pill testing service was provided [26].

At its core, ‘pill testing’ enables a potential consumer of unregulated drugs the time to pause, test the product which they have possession, and discuss the results with multi-disciplinary and non-judgemental health professionals, prior to ingestion. The term ‘pill testing’ is colloquial and used by the authors as it is the most commonly used and properly understood term used in Australia for ‘drug checking’ (see Fig. 1).

**Fig. 1:**
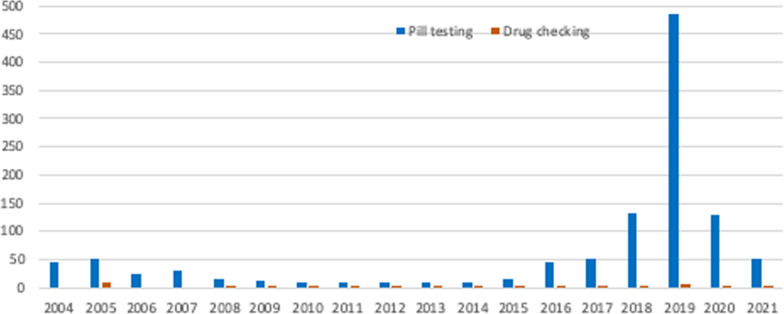
Google mentions of 'pill testing' versus 'drug checking' in Australia, from 2004 to 2021

## Approach

Theory of change models is common in the wider world of management theory [5, 21]. One that is well-described is Kotter’s 8 Step model [22] which has increasingly been cited by health-based teams as providing a strategic template for health policy reform [44]. Although the original model was proposed to be sequential between steps, with some overlap, it has been suggested that its application in health could also be iterative in application. Moore, Yeatman and Pollard [31] offered 2 additional steps in their otherwise complementary analysis. We use Moore, Yeatman and Pollard’s 10 Step Change Model (see Table 1) to outline the activities that lead to the introduction for the first legally sanctioned pill testing trial. The paper takes each step of the model and illustrates the key activities that largely occurred over an approximately 24 month period resulting in the service being operational on 29 April 2018.

**Table 1:** Phases of the step change model

Step	Phases	The 8 phases of Kotter’s 8 Step for Leading Change	“Kotter Plus – a 10 Step Plan”:
Step 1	Establishing a Sense of Urgency	✓	✓
Step 2:	Creating the Guiding Coalition	✓	✓
Step 3:	Developing and Maintaining Influential Relationships	**□**	✓
Step 4:	Developing a Change Vision	✓	✓
Step 5:	Communicating the Vision for Buy-in	✓	✓
Step 6:	Empowering Broad-based Action	✓	✓
Step 7:	Be Opportunistic	**□**	✓
Step 8:	Generating Short-term Wins	✓	✓
Step 9:	Never Letting Up	✓	✓
Step 10:	Incorporating Changes into the Culture	✓	✓

Source: Adapted from Kotter [22] and Moore et al., [31]

Our focus is on examining the strategy and activities of the small group of actors who were involved throughout the 24 month period in establishing the service. We have not sought to interview those observing the process who were not party to decisions or in the room at the time. In one sense, this could be seen as a collaborative auto-ethnographic study [40] as it reflects our subjective perspectives as insiders to the process.

As such, it relies on a chronology of events, public information and personal and memory data or narratives. Noting that memories are constructed and modified over time, we have used the time stamp available from media campaigns, music events and written correspondence to provide a timeline of meetings and key events leading up to the first pill testing service. Not every one of us attended every meeting or was party to every activity across the period. To be able to elaborate the actual strategy and specific approaches applied over this period, we accessed multiple sources of qualitative data.

This data can be grouped into two categories—internal and external. Internal data included personal calendars, email and text correspondence, and key players recollections of events and conversations. External data include notes and minutes from meetings, press releases, news stories and media interviews conducted with key players.

With multiple sources of data on the same phenomenon across each of us it was possible to triangulate our perceptions on a particular event or strategy and to cross-reference with the external data to collaborate the description, we have provided of the events.

We have then used the phases of the step change model to thematically group our qualitative data. Activities or strategies do cross over into two or more steps of the change model. Although the step model is linear as noted above, there is some iteration that occurs over time. We have used our interpretive judgement to decide where an activity is best placed into either a single step or to refer to the activity in one or more steps.

A limitation to this approach is that the strategies and activities are focussed on the specific establishment of the first legally sanctioned pill testing service and does not account for other concurrent developments relating to pill testing services in Australia that may have been occurring. Nor do we account for how those who were either observing our activities, or were ‘recipients’ of our activities, interpreted those activities.

However, we feel it is important to document the inside workings of a strategy that has had real success. We would posit that academic papers on the lived experience of the activists and advocates and the leadership required when working in a contested space is of benefit to those contemplating their own reform agenda. It also provides a unique case study to assist in teaching.

## Step 1: Establishing a sense of urgency

Large-scale dance music festivals, or ‘raves’, had arrived in Sydney and Melbourne in the late 1980s [25]**.** These were largely underground events in warehouses and fields where alcohol was not sold. As the rave scene evolved it moved to licensed clubs and commercial development of large-scale festivals where alcohol was sold. The increasing regulatory controls over licensed premises and events in relation to alcohol complicated possible responses to reduce the harm from designer drugs, which is still a complicating factor today.^1^ At the time, the various regulatory controls in the major cities also lead to the emergence of a vibrant outdoor culture, sometimes locally called ‘bush doofs’ [25]. With different and stronger forms of new designer drugs emerging at an increasingly rapid rate, people using these substances had introduced rudimentary testing processes [13].

By the 2010s, more dangerous substances were emerging exponentially, and MDMA availability and use at festivals were increasing. Growing numbers of young people were now dying or suffering life threatening overdoses, of varying provenance, at music festivals across the country [16, 30, 38, 41, 43, 46]. The mechanisms put in place to address drug consumption in Australia (sniffer dogs, strip searching) were failing, being simultaneously inappropriate, counter-productive and arguably unethical [17] and even causing unintended additional harm [28]. A growing number of young Australians, both those who used and did not use drugs, realised that another approach was needed. Perhaps as importantly, so did their parents.

A coalition of individuals and organisations who were calling for drug reform and implementation of new harm reduction services such as drug consumption rooms and pill testing was emerging. They utilised a range of tactics to build a sense of urgency. Key advocates such as Matt Noffs (CEO of the Ted Noffs Foundation), Gino Vumbaca (President of Harm Reduction Australia) and David Caldicott (an emergency medical consultant), amongst others, were highly visible across the traditional and social media outlets. They supported coronial enquiries in NSW and lobbied politicians and advisors in various state and territory governments to consider drug reform. Noffs published two books targeted at ‘mums and dads’ to influence their thinking and suggesting viable options for changing the policy narrative [34, 35].

On 2 March 2016, the cross-party Parliamentary Group on Drug Policy and Law Reform held a one-day drugs summit in Canberra, the outcome of which was The Canberra Declaration. Advocates were able to secure the adoption of the following recommendation: *‘Drug checking presents as a potentially valuable option for reducing harm at public events and governments should enable trials to be implemented as a matter of priority’* (Australian Parliamentary Drug [7].

As the public debate became more mainstream, the importance of the problem under consideration was evident and change was both ‘necessary’ and ‘achievable’ [18].

### Step 2: Creating the guiding coalition

In the early and mid-2000s activists, such as Caldicott, then in South Australia, attempted to establish a pill testing service in that jurisdiction [13]. Despite extensive and intensive attempts during this period to engage politicians, policy makers and researchers in South Australia, there was no appetite at the state level for such a service. This was mirrored in other jurisdictions across Australia at state and federal level. Furthermore, there was not a sense of urgency that something needed to be done; little was known about the use of pills at festivals in the broader community, and it was not seen as a ‘problem’. Despite this, these formative years were responsible for establishing many relationships that underpinned the emergence of Pill Testing Australia.

By the mid-2010s, the prospect of establishing a guiding coalition in the ACT to bring about a government sanctioned pill testing service seemed like a realistic prospect. The ACT was a small ‘l’ liberal jurisdiction with a Labor Government, in coalition with a significant Greens minority, making it one of the most progressive jurisdictions in Australia [29]. Shane Rattenbury, leader of the ACT Greens, was a powerful advocate for change. Coupled with the sense of urgency generated around overdoses, deaths, and the inability of police to effectively address drug use as a law enforcement issue, the precursor conditions to enable a stable and sustainable coalition to be built had coalesced in time and place.

Matt Noffs proactively reached out to Gino Vumbaca and David Caldicott to discuss how they could build such a coalition to both change government policy and deliver pill testing at festivals. After several meetings in Canberra, the first formal meeting of this group was convened on 15 December 2016 at the Noffs’ ACT facilities. The Safety Testing Advisory Service at Festivals and Events (STA-SAFE) consortium was convened under the auspices of Harm Reduction Australia, led by Vumbaca. The broad agreed principles of the group were:

- ‘Project’, before ‘personalities’ or ‘publications’
- Chatham House rules for any discussions
- A shared policy vision including that the service be sanctioned by government
- A shared implementation strategy including that the service would be a health-based model
- A commitment to the collection of operational data to inform better policy and practice.

The initial coalition consisted of a clinical lead, a research lead, leading advocates from the alcohol and other drug (AOD) sector and representatives of users of the service. Managing different agendas and personalities can present significant challenges to maintaining focus on the core outcomes. David et al. [18] identify some of the challenges associated with the creation of coalitions, including disagreement over the end goal or how best to achieve the goal, poor dynamics with the coalition, a competitive environment where individuals are more focussed on their work than the end goal, limited financial resources, lack of clear roles and inability to sustain long-term partnerships. A need to rely on in-kind contributions and volunteerism, as well as the oft-misunderstanding of harm reduction interventions and the stigmatisation of drug use, were also significant challenges faced by the group.

The careful selection of the group membership and the articulation of the end goal, strategy and agreed principles mitigated against potential issues identified by David et al. [18]. Roles, responsibilities and expertise were clear with overall leadership and co-ordination being led by Vumbaca. Financial constraints were overcome by significant in-kind support, hundreds of pro-bono hours of planning and meetings, and key donations by the Noffs Foundation and Harm Reduction Australia to support the pill testing activities.

## Step 3: Developing and maintaining influential relationships

Although the guiding coalition was a small group, they reached out to recruit key people with specific skills in public policy and media advocacy, emergency medicine, evaluation and research, risk management, and peer counselling. Alongside this was a strategy of developing influential relationships critical to achieving success.

Unlike many jurisdictions in Australia, the ACT’s main analytical laboratory was under the aegis of ‘health’ not ‘law enforcement’. This allowed the rapid analysis and identification of substances deemed to be of public health concern via admissions to emergency departments of suspected overdoses, which was initiated by the ACT Investigation of Novel Substances (ACTINOS) Project [14]. This led to the ACT Chief Health Officer (CHO) becoming increasingly interested in festival-based pill testing, and he initiated a dialogue with other key institutional players. Key amongst them was the ACT Chief Commissioner of Police, with conversations occurring with the group in August 2016. An initial meeting with Caldicott turned into several with senior police executives and then, local area commanders. Frank Hansen, a former senior NSW police officer and long-term chair of the Intergovernmental Committee on Drugs, Chair of the Noffs Foundation Board and member of the Harm Reduction Australia Board, was active in talking to ACT Police. Emerging support of senior law enforcement enabled engagement with elected representatives, who were as yet uncommitted to the process. Law enforcement support has been identified as an important component of any drug law reform in Australia [24].

Caldicott presented on pill testing at The International Association of Forensic Toxicology meeting in Brisbane in August 2016, and this led to the relationship between the consortium and FACTA (Forensic and Clinical Toxicology Association), the peak body of analytical toxicologists in Australia. Shortly after, an invitation to present at the United Nations Office of Drug Control in Vienna also allowed international partnerships to be formed. These in turn were used to introduce the CHO to Europeans involved in pill testing when he travelled there and later the Europeans hosted our chief chemical analyst for his Churchill Fellowship.

The meaningful involvement of peer workers is crucial for the success of any harm reduction service. Where the service delivery setting is a music festival, it is beneficial to engage festival harm reduction workers, who are familiar with the work environment and cohort of festival patrons [47]. David Caldicott had met Harm Reduction Victoria (HRVic) DanceWize Program Coordinators at a conference in 2014 where they presented on the demand for pill testing services. The PTA coalition’s relationship with HRVic’s Dancewize and later other festival-based and/or youth orientated harm reduction initiatives like Students for Sensible Drug Policy Australia (established in 2016) and NUAA’s DanceWize NSW (established in 2017) was critical to the coalition being able to provide a service both in terms of awareness-raising and practical logistics. Jenny Kelsall, HRVic CEO until 2017 and a progressive-thinker, allowed DanceWize’s onsite data collecting templates and outreach inventory to be used, and DanceWize staff and a team of Key Peer Educator and Carers to volunteer at the 2018 and 2019 PTA pilots [48].

Simultaneously, the various medical colleges and professional medical bodies were beginning to approach Caldicott and other medical practitioners to discuss their positions on the issue. Over time, and after many hours of consultation, a series of supporting positions were elicited. A process at the end of which saw more than a dozen professional bodies as supporters (see Table 2 for examples).

**Table 2:** Examples of position statements and major press releases by key health bodies

College/body	Date	Weblink
*Position statements*		
Public Health Association of Australia	2017	https://www.phaa.net.au/documents/item/1955
Pharmaceutical society of Australia	2019	https://my.psa.org.au/servlet/fileField?entityId=ka17F000000cmhbQAA&field=PDF_File_Member_Content__Body__s
College of Emergency Nursing Australasia	2019	https://www.cena.org.au/public/118/files/Governance/CENA-Position-Statement-Pill-Testing.pdf
Australian College of Rural and Remote Medicine	2022	https://www.acrrm.org.au/docs/default-source/all-files/college-position-statement-pill-testing.pdf?sfvrsn=22676dec_15
*Major press statements*		
Australasian College for emergency Medicine	2019	https://acem.org.au/News/Jan-2019/ACEM-supports-the-introduction-of-pill-testing-tri
The Royal Australian and New Zealand College of Psychiatrists	2019	https://www.medicalrepublic.com.au/pill-testing-passes-festival-test-flying-colours/20724
Royal Australasian College of Physicians	2019	https://www.racp.edu.au/news-and-events/media-releases/racp-writes-to-nation-s-leaders-there-is-sufficient-evidence-to-support-pill-testing-trials

There were also thousands of hours of back door conversations with key influential people across the political spectrum, in both state and federal politics, public servants and media organisations. The purpose of this was not necessarily to try and get them to provide ‘public’ support, but to provide support behind closed doors, or to not actively *oppose* reform, which can be just as important. Where party loyalty is a strong aspect of politics in Australia, individual ministers might support a particular initiative but if it is not party policy, it is highly risky for them to go public. A very typical and explicit refrain was ‘Of course, on a personal level, we completely support you, but we couldn’t possibly support it publicly’. Similarly, police and health officials cannot publicly contradict the government of the day on policy, but they can be neutral in the closed discussions, or even be powerful advocates. The consortium developed and maintained such bipartisan links across political parties, law enforcement officers, health officials and the media.

## Step 4: Developing a change vision

One of the lessons learned from early in South Australia was that the appetite in Australia for a European-style pill testing program was simply not there—it would need to be an *Australian* model, designed for the Australian socio-political *terroir*, to have any chance of local success. Following a series of face-to-face meetings, email exchanges and discussions five elements to our approach emerged:

- The service would be government approved and include medical oversight
- A charter would be developed that would provide an ethical platform upon which we would base our practice
- A rigorous analytical process for the chemical testing that met all of the requirements, and where possible exceed the expectations of the analytical critics
- Only professionally qualified volunteers would be used in all areas of service delivery
- Timely collection of data to inform a credible and publicly available review of the operation of the pill testing service.

The Charter was a code of conduct developed to articulate the principles that PTA and other aspiring providers would advocate for and adhere to (see Table 3). As New Zealand were also advancing in their efforts to bring about pill testing, their lead agency The New Zealand Drug Foundation, through their then leader, Ross Bell, lead to the charter morphing into the Trans-Tasman Charter (TTC) in August 2018 [39]. Through advocacy, more than a dozen service providers signed up to the charter.

**Table 3:** Principles underpinning the Trans-Tasman charter

Principle	Explanation
Front of House Testing	Ensuring a real-time face-to-face interaction between those testing pills and those presenting their pills for testing within a harm reduction framework
No Fee for Service	Delivery as a free universal public health and harm reduction measure for those accessing the service
Peer Driven	Should involve peers and young people in all stages of its design, development and delivery
Information Sharing	To expand and share evidence informed public health and harm reduction policies and programs
Open Science	A commitment to publish scientific research, data and dissemination via open access journals and archives, advocate for the scientific process, and communicate scientific knowledge independent of political imperative

One of the major objections deployed to block the advancement of a pill testing service was the focussed opposition from a small number of credible doctors and chemical analysts. The argument was that pill testing was not technically capable of providing accurate results in real-time at a live festival or would do more harm than good [42]. One challenge was that were no ‘Australian Standards’ for pill testing at music festivals, unlike those that existed for urinalysis to determine cut-off levels for a positive result [26]. There was also the issue of new drugs emerging that were not in the analytical libraries that are used to assess the likelihood of the presence of particular compounds, and potent compounds that could exist at very small doses, whilst still being pharmacologically active, might not be detected. Another issue was the capacity to conduct confirmatory analysis on site at the festival.

The reliability and validity of the chemical analysis was critical to the credibility of the service. It was determined that the analysis would be performed by analytical chemists, using equipment vetted by them. This is different to the process used in some jurisdictions overseas. The consortium expanded early on to include a team of analytic chemists from the Australian National University, led by Malcolm McLeod. On 16 June 2016, Mal McLeod, initially a sceptic, travelled to Calvary hospital to meet with Caldicott and run the first samples (salt and morphine) on the work horse of the process, the Bruker alpha FTIR. A few years later, following the FACTA conference in Adelaide in June 2019, a robust debate resulted in the formation of a task force within the lead agency to examine best practice for pill testing in Australia. The debate within analytical circles was no longer ‘whether’ pill testing should be provided, but the best way to provide it and the need to develop agreed professional standards.

## Step 5: Communicating the vision for buy-in

Public opinion can and does influence government policy and politicians [29]. The Overton window has been used to describe how and when politicians feel able to support particular policies [6]. In this approach, the first step requires changes in broad public opinion about what is politically achievable. However, this required effective communication to ensure that as many people and groups understood the vision for change and why this particular strategy, pill testing was the one to support over the alternatives. The assumption that the passive creation and provision of erudite research alone would result in change, particularly in such a contested political and social space, was wishful thinking.

Vumbaca and Caldicott were experienced in dealing with the media. Noffs provided a further range of media contacts and a more targeted strategy to maintain the sense of urgency for change. Influential individuals were deployed to write opinion pieces in major newspapers and the issue began to be aired on key discussion forums such as The Project [32]. A key component of the news strategy was to also engage with the conservative media so as to influence both conservative politicians and conservative voters who were most likely to be opposed to harm reduction strategies. In December 2016, Noffs had launched the ‘Time to Test’ campaign that then morphed into the ‘Take Control’ campaign in 2018 [45].

These members of the group were effective ‘change champions’ who expressed their passion for change, often robustly. Such advocates are an essential component in generating interest and support for an issue; it is also that single minded passion that galvanises them to pursue their goal even in the face of negative and sometimes destructive commentary.

The adopted strategy fulfilled the general principles of Dorfman and Krasnow [20] for public health communication. Table 4 outlines the ‘who’, the ‘what’ and the’ how’ of principles that should underlie any health communication strategy. The consortium was clear that media activities (both traditional and social) were focussed on getting strong support for the vision, by both the general public and politicians. It was constantly affirmed that the service would have medical oversight to increase confidence in the delivery model. This was considered essential to get buy-in by the wider community, medical practitioners, and eventually influential political operatives.

**Table 4:** General principles of public health communication

General principles	Principles specific to pill testing
Who was the message for?	General public and politicians
What was the message being communicated?	This is a health and medical intervention with highly professional clinical, analytical and peer staff. There is an evidence base to support the intervention. The service has been successfully deployed in many other countries
How was the message being communicated?	Directly to target groups, and not through academic publication or committee meetings. Specific media outlets were identified and a strategy devised to engage them in the process of communicating the service

Source: Adapted from Dorfmann and Krasnow (2014)

An important aspect of the communication strategy was to provide a counter narrative to the fallacies and alternatives propagated by opponents. Table 5 outlines the major narratives that the consortium confronted, where countering these relied on communicating the known evidence base around pill testing and drew on the implementation of similar services overseas, that both Caldicott and Noffs had visited on separate occasions.

**Table 5:** Communicating counter narratives to pill testing

Arguments against pill testing	Counters
Green light for drug use	People are about to consume a drug and pill testing provides a moment of pause and discussion with qualified health professionals
Inaccurate information	Highly technical equipment used by credentialed analysts
Insufficient information	Articulation of the public health principle that the more information a person has the better their decisions are likely to be
Already effective policies in place	The continued tragic deaths at festivals, as investigated by Coroners, showed current policies more likely to increase rather than decrease harm

Using the media ‘for good’ has the effect of acting as a force multiplier. It vastly increases the availability of the message beyond the horizons of those relying on academic journals, whilst both directly and indirectly targeting influential individuals and groups. Social media had a further catalytic effect, particularly amongst non-academic supporters, galvanising the movement with the specific aim of creating dinner party conversations on the issue or, in the Australian vernacular, ‘barbeque stopper’ conversations.

## Step 6: Empowering broad-based action

Copies of relevant information were circulated to all members of parliament, federal, state and territory throughout Australia, as well as other key stakeholders. The dissemination of information to parliamentarians continued throughout the process and continues. In the ACT, a full proposal outlining all the operational and procedural elements of a pilot festival-based pill testing service was developed by the consortium, with input from Matt Harper, from Tigertail Risk Management, and presented to the ACT government. This provided opportunities for a series of meetings with ministers and key officials where *all* aspects of the proposal were scrutinised and discussed. At one stage that extended even to the colour of the uniforms of potential service providers.

Ministers rely on the advice provided to them by their public servants. As already noted, the consortium sought to engage public health and law enforcement officials so that, at worst, they would be neutral and, at best, supporters of reform. As a key principle was to have a government sanctioned pill testing service, these people were critical. Both direct and indirect means were used to engage key officials in the discussions.

Another key groups are advisors in the ministers’ offices. They have multiple roles, including influencing ministers on ‘good ideas’, encouraging public servants to deliver on the ministers’ ‘good ideas’, and coordinating across multiple departments to achieve support at cabinet level for the ‘good idea’. From the early days, Vumbaca built trusted relationships with ministers’ offices as without the support of cabinet; there would be no pill testing. It was also an important mechanism to head off any emerging obstacles from within the bureaucracy.

As part of the process to remove barriers, the consortium sought to remove any financial impediments by offering to deliver the festival pill testing pilot program completely free of charge, with all costs, including equipment, insurances and volunteers, to be met by Harm Reduction Australia. Some argued this was not a sustainable model going forward but our view was that it was necessary to demonstrate the service could be provided safely in the first instance. Then, funding models might be more forthcoming.

As the momentum for change gathered pace and individuals could see, there was a ground swell of support exhibited by social media campaigns, trade magazines running pro-pill testing campaigns, and barbeque stopper discussions, the consortium provided opportunities for those who wanted to be involved to do so. Events at the Victorian State Parliament and Sydney City Council, as well as at Parliament House in Canberra, included parent advocates who had lost loved ones to talk about their experience. Young people were engaged through student advocacy groups such as Students for Sensible Drug Policy.

Skilled and experienced supporters, including counsellors, chemists, doctors and peer workers came forward, offering to provide assistance.

However, building a highly public action base had to be tempered with the understanding that many, particularly, some parents and families were acutely affected and traumatised by the loss of loved ones. Our team reached out to those deeply affected to privately offer solace and comfort, as well as engaging the support of services such as Family Drug Support to assist. There were never any requests, or expectations, of grieving relatives to become involved in the process, but the support extended often led to parents and family members becoming some of our strongest and most important advocates.

## Step 7: Be opportunistic

To facilitate the commencement of pill testing also meant seizing every opportunity to promote the program and its benefits, every media opportunity was accepted, including with opponents in the media. Consortium members gave lectures on drug policy to university students, spoke to high school groups and answered personal enquiries by school children doing projects on pill testing. No request was too minor to be addressed. At times, inquiries and offers to assist overwhelmed the capacity of the group to deal with them all and on occasion that caused resentment with those trying to help. Consortium members were summonsed to coronial inquiries, including giving expert witness statements, made submissions and engaged with coronial and judicial officers to visit festivals where the opportunity arose.

The availability and cost of the testing equipment were a significant hurdle. Noffs introduced Vumbaca to a contact at Bruker Corporation to see if they would loan the testing equipment to the consortium. This provided Bruker with the opportunity to test their libraries in a ‘real-time’ environment whilst providing the service with high quality equipment at no cost. Noffs drove from Sydney to Canberra carrying the equipment.

There were presentations at various conferences and seminars to promote the vision and strategy [10, 11]. There was active engagement across a broad range of social media outlets that continued post the 2018 pilot including Q&A [1], 730 Report, [2] and 60 min [33].

## Step 8: Generating short-term wins

The successful implementation of the 2018 pilot pill testing service at *Groovin the Moo* Canberra was a highly prized short-term win in a longer-term strategy. A successful pilot and the important service report generated by the consortium [27] provided new opportunities to gain further wins. The operational review of the service was considered a key plank in the strategy to build momentum for longer-term structural reform. It was critical to produce a credible evidence base outlining the design of the intervention, collection of data to inform the report, and production of a document that was timely and useful.

The service provided the opportunity to collect high quality data from client users and link that to the chemical analysis. The latter was particularly important as forensic data on what the substances actually contained, and whether it matched what individuals thought they were taking is of considerable interest to the media and the wider community. Analysis of the data found ‘only 43% agreement’ between what people thought they were taking and what was in the drugs. This demonstrated ‘that patron’s knowledge of what they are taking is often not well founded’ [27]. The actual test results demonstrated that the technology could be both effectively deployed in a festival environment and identify drugs of concern.

*N-ethylpentylone, which is a synthetic cathinone, that has stimulant effects and significant neurotoxic potential. It is active in low doses (1 mg) and has been documented for causing fatalities and other harms, as an adulterant in commonly sought substances like MDMA.*

*One case of N-Ethyl pentylone, which is a significantly more potent than MDMA with a high risk of death from microscopic amounts, was detected on the night.*

Following counselling after receiving the results of the chemical analysis, 42% of participants intended to change their behaviour based on the medical and counselling advice they received at the service, demonstrating that the service had the potential to affect behaviour [27]. This was later confirmed by the evaluation of the 2019 service [36, 37].

The consortium had the evaluation and research skills to put together a data collection plan that would not impede service delivery and was supported by counsellors, as they would need to administer short barrages of questions to clients. This required flexibility by the volunteers from DanceWize and was critical to delivering the service report. Although a relatively small number of cases, the quality of service delivery and data was the more important factors in the first pilot. Importantly, 74 of the 83 people who utilised the chemical testing on the night agreed to provide the necessary information to enable the operational service report to be written.

This demonstrable short-term win was crucial to recruiting more professional bodies as supporters of the cause. Coronial investigations and other inquiries were able to utilise the service report to bolster their findings and recommendations to governments. On the night, key stakeholders, including the Minister, senior police and ambulance officers and media representatives, were able to see in real-time the professionalism of the model that the consortium had developed and the benefits of the service in action. Much of the methodology was replicated for the evaluation of the 2019 GTM festival testing service [36, 37] and later employed in the fixed pill testing trial.

There was now majority support across the community, and particularly in the ACT, supporting pill testing by the end of 2018. Table 6 shows that support was regardless of political preference, although conservative voters were somewhat less likely to support pill testing. Males and females were equally supportive and, predictably, younger people were more in favour, although 55 percent of people aged 55 or older were supportive. It was also evident that people had a view, with only 25 percent, indicating that they were not sure. By the time of the ‘Australian Election Study’ later in 2019, only 15 percentage did not have a view [29]. This suggests that the messaging around the need for a service and the supporting evidence base had crystallised in most citizens minds with the majority supporting the service.

**Table 6:** Support for pill testing in December 2018, column percentages

	Total	Voting preference				Gender		Age group		
		Labor	Lib/Nat	Greens	Other	Men	Women	18–34	35–54	55 +
Support	59	63	57	78	54	58	60	67	54	55
Oppose	17	13	21	6	23	20	13	12	17	21
Do not know	25	24	21	16	22	22	27	21	29	25

Exact wording: Thinking about drug policy, do you support or oppose pill testing services (where trained counsellors provide risk reduction advice informed by on-site, laboratory analysis of people’s drugs)?

Source: https://essentialvision.com.au/pill-testing, accessed 1 Feb 2023

This increased public support was confirmed by the ‘Australian Survey of Social Attitudes’ (AuSSA), which is Australia’s main source of annual data for the scientific study of the social attitudes, beliefs and opinions of Australians. Seventy one percent nationally supported pill testing in both festivals and health settings. Those who did not know had dropped by half from 2018 to 202/21to 13 percent.

Short-term ‘wins’ also provided the impetus to achieve longer-term goals and reinforce the change vision. It allowed for a building of momentum and communication to a sector and base desperate for reform but starved of wins. It energised and galvanised efforts as more people became optimistic and supportive about change.

## Step 9: Never letting up/focus

Accepting the support and enthusiasm of a wide range of parents, families, people who use drugs, advocates and some key politicians and officials was important in building the environment for change in the two years prior to the trial. However, this has been a campaign 25 years in the making, with a second sustained drive for reform from 2015. Success was achieved by focussing on the sole end goal of delivering an early intervention service to inform, educate and encourage drug users, new and seasoned, young and old, to make wiser choices.

The capacity of the group to be flexible and opportunistic came to the fore when two earlier festivals appeared to be likely possibilities, but service delivery did not eventuate. The first was the intention to deliver the service at *Spilt Milk* in November 2016 and efforts by Tzanetis, Caldicott and others were hopeful until the week prior to the event [15]. Then *Groovin the Moo* in April 2017. This did not proceed as it was alleged that the consortium had not compiled the documentation required by the ACT government and other relevant parties. This was disputed, as the exact nature of the documentation required by government was never fully articulated, and the demands were made only in the week preceding the event. Regardless, it was early days in the group formulating a coherent service delivery model with all of the risk management structures and protocols required. As a small group with limited resources, it took time to develop the basic infrastructure to support such a service. It was also early days for the event organisers who were also working out their protocols for such a service.

Despite this initial setback, the group continued to refine its processes, procedures, and, more importantly, maintain close links with the ACT Government to ensure that all the issues were dealt with. In September 2017 the ACT Government indicated that they were now satisfied with the STA-SAFE proposal. All key stakeholders, including the health department and law enforcement, were satisfied with the protocols and had developed their own operational procedures for the event. The next festival was the *Spilt Milk Festival* in November 2017. One remaining issue was that the festival was being held in Commonwealth Park, which is managed by the National Capital Authority (NCA) on behalf of the Australian Commonwealth, not the ACT government.

Following submission of the paperwork which met ACT government requirements, *Spilt Milk* organisers advised the consortium that the NCA required additional documents on the Wednesday before the festival. Again, the nature of those documents had not been articulated, but the more important aspect was that it would take time for the NCA to approve the additional document, putting at risk the whole festival. The ACT Liberals, who opposed the trial on principle, had indicated the land was controlled by the Commonwealth (also a Federal Liberal Government, who were also opposed to any form of pill testing) and that it was unlikely to be approved. The ACT opposition minister for health had foreshadowed this tactic several months earlier, and the leader of the opposition wrote to the relevant federal minister to request that approval be denied. This was achieved. Within 24 h, we were informed that the deadline for the additional documentation had passed.

We then focussed on the next major festival in Canberra—*Groovin the Moo* in April 2018, which was *not* on Commonwealth land. The group continued to ensure pill testing remained as a high-profile issue in the wider community and the media. We continued advocating, leveraging the various relationships that had been built over the previous 18 months. There was still a great deal of nervousness about how the pilot would go, and how it would be accommodated within the venue. There was also an attempt to control who in the STA-SAFE consortium could be present at the site on the night. In the end, the key players in the group refused to agree to that level of control. Agreement between STA-SAFE, the ACT Government, the University of Canberra, as the landowner, and the festival promoters was reached three days before the festival was to commence.

Pulling together all the team members, volunteers, insurances and resources required to undertake the pilot, as well as accommodate the restrictions of promotion and advertising imposed as part of the approval, required a high level of flexibility, effort and mobility. This flexibility to adjust to new requirements was possible as a cohesive team with a clear strategy had been built over the previous 24 months.

## Step 10: Incorporating changes into the culture

Delivering a successful service in the festival environment re-enforced with the bureaucracy that change was possible and that risks could be managed. The strong community support for pill testing in the ACT also emphasised to local politicians that this was a popular measure. It became an innovation that the ACT could be proud of and, as a result, it was relatively straightforward to run the service again the following year.

Lessons were learnt and applied. An experiment to have those who were tested carry a wrist band with details in case they ended up in hospital was found to have low compliance and was not used in the 2019 service. Improvements were negotiated with the organisers to increase the space in 2019. Following the publication of unauthorised photos by *The Australian* that potentially identified individuals in 2018, the 2019 service held a special event prior to the day to let the media through. This same strategy was used when first opening the fixed pill testing site. It was evident that two machines would be required for the 2019 service and Vumbaca took out a personal loan to secure it. Later a philanthropic organisation came forward and reimbursed Vumbaca.

Further innovation on the analytic techniques by the chemists were implemented and ties were built to other chemists to improve the standards around testing. ACT Health released a specific festival pill testing policy (ACT [4]. There was on-going media engagement and presentations at various venues to promote the benefits from the trials [12, 49].

Following the success of the festival testing process, a local service provider, Directions Health’ and Pill Testing Australia began discussions in May 2019, with a view to developing a proposal for the current fixed site drug checking service for Canberra. The first meeting was in July of that year, in parallel with the successes of the festival-based programs, with Pill Testing Australia providing the analytical equipment, and chemical and clinical support to the service hosted by Directions Health, and include CAHMA (the ACT peer-based organisation for people who use drugs) as partners. The success of the service to date has solidified the concept of pill testing in the ACT.

## Conclusion

The Kotter/Moore Model has provided an analytic tool to describe the factors that have come together to result in the first legally sanctioned pill testing service in Australia. Although the model has been applied retrospectively as a heuristic device, it suggests that the framework might be useful to proactively deploy by others considering the implementation of contested policy in the AOD sector. A key challenge to utilising the framework is to recognise where activities cross ‘steps’. The utility of the framework is in identify the key aspects and at what stage of the strategy there needs to significant focus on that activity. A second key challenge is to not interpret the steps as a formulaic linear set of activities. Implementation is messy and often complex, so we would argue the model provides a useful framework for those leading the proposed reform to structure strategy and activity recognising that there are feedback loops and changes that occur as the implementation progresses.

The actions and interventions might be considered as coming from *outside* the traditional ‘establishment’ of drugs policy in Australia, as it drew heavily on service providers, the users of drugs, and the families of users. Those involved in the process were less unencumbered by the shackles of those with closer ties to funding bodies and agencies. They were able to engage with the general public in non-traditional ways, through digital platforms, social media, direct public action, and direct political engagement. On many occasions, colleagues from other agencies expressed admiration and regret that they did not have the leeway to take similar approaches in affecting change.

At the time of writing, the government of Queensland has committed to a service citing the successes of the ACT as one of their inspirations. It is still not all plain sailing for pill testing in Australia [9]. Australia’s largest State, New South Wales, is still prevaricating, with a promise to reconsider at a drug summit. However, multiple other jurisdictions have reinforced their opposition to pill testing in the light of QLD’s recent announcement [3].

Festival-based testing in Australia has encountered a further roadblock in the shape of sudden and vastly over-inflated insurance premiums for providers, and the festivals at which they operate. This is despite the entirety of available evidence that demonstrates that such services can reduce the harms associated with drug consumption at music festivals. This is not a problem suffered by our nearest neighbours in New Zealand.

Although pill testing is just another health intervention, what it represents is a challenge in direction of 100 years of drug policy which has been dominated by a largely prohibition model. There have been some hard fought harm reduction measures introduced along the way, but pill testing is fundamentally different from many of these harm reduction services as it is targeted at a much wider population than injecting drug users or those with serious drug dependencies. It encompasses many young people and their parents, many of whom have been exposed to drugs during the latter part of the 20th and early twenty-first centuries. As a result, pill testing represents a shift in core social values about drug use and thus remains highly contested in Australia.

## Data Availability

Data referenced in this paper are cited from open sources.

## Footnotes

1 We are grateful to one of the reviewers for pointing out the complication of alcohol in providing harm reduction services.
